# The Tracking of Speech Envelope in the Human Cortex

**DOI:** 10.1371/journal.pone.0053398

**Published:** 2013-01-10

**Authors:** Jan Kubanek, Peter Brunner, Aysegul Gunduz, David Poeppel, Gerwin Schalk

**Affiliations:** 1 Department of Anatomy & Neurobiology, Washington University School of Medicine, St. Louis, Missouri, United States of America; 2 Department of Biomedical Engineering, Washington University in St. Louis, St. Louis, Missouri, United States of America; 3 Department of Neurology, Albany Medical College, Albany, New York, United States of America; 4 Brain-Computer Interface Research & Development Program, Wadsworth Center, New York State Department of Health, Albany, New York, United States of America; 5 Department of Psychology, New York University, New York, New York, United States of America; University of Barcelona, Spain

## Abstract

Humans are highly adept at processing speech. Recently, it has been shown that slow temporal information in speech (i.e., the envelope of speech) is critical for speech comprehension. Furthermore, it has been found that evoked electric potentials in human cortex are correlated with the speech envelope. However, it has been unclear whether this essential linguistic feature is encoded differentially in specific regions, or whether it is represented throughout the auditory system. To answer this question, we recorded neural data with high temporal resolution directly from the cortex while human subjects listened to a spoken story. We found that the gamma activity in human auditory cortex robustly tracks the speech envelope. The effect is so marked that it is observed during a single presentation of the spoken story to each subject. The effect is stronger in regions situated relatively early in the auditory pathway (belt areas) compared to other regions involved in speech processing, including the superior temporal gyrus (STG) and the posterior inferior frontal gyrus (Broca's region). To further distinguish whether speech envelope is encoded in the auditory system as a phonological (speech-related), or instead as a more general acoustic feature, we also probed the auditory system with a melodic stimulus. We found that belt areas track melody envelope weakly, and as the only region considered. Together, our data provide the first direct electrophysiological evidence that the envelope of speech is robustly tracked in non-primary auditory cortex (belt areas in particular), and suggest that the considered higher-order regions (STG and Broca's region) partake in a more abstract linguistic analysis.

## Introduction

Spoken language is central to everyday communication. How speech is represented and processed in the nervous system is therefore of considerable interest to a wide range of scientists, clinicians, and engineers.

Traditionally, the speech signal–-an output of the vocal tract resonating at specific frequencies–-has been viewed as a time-varying sonographic pattern of information in the frequency domain [1]. Recently, much interest has been sparked by purely temporal features of speech [2]–[5]. The importance of temporal information is demonstrated by many cases of language impairment [3]. For instance, auditory neuropathy distorts temporal information transmitted to the brain. Patients with this condition can hear common sounds, but are severely impaired in understanding speech [6]. Other clinical evidence further supports the critical importance of temporal information. For example, early models of cochlear implants stimulated the cochlea with just one channel. This way, speech information was delivered to the brain entirely in the form of a time-varying waveform. Yet, patients with such implants were capable of understanding speech surprisingly well [2].

These salient clinical cases have stipulated questions about which component of the temporal signal is essential for speech understanding. Particular attention has focused on the slowly varying temporal component of speech (“envelope,” also referred to as “amplitude-envelope,” “time-amplitude,” or “time-intensity” [2]). Two main streams of evidence fuel this interest. First, it has been shown that manipulations of the speech envelope affect the recognition of consonants, vowels, and the understanding of sentences [7]–[9]. This evidence indicates that speech envelope is an auditory feature that is necessary for speech understanding. Second, it has been shown [10] that human subjects can understand speech with a preserved temporal envelope but with severely degraded frequency content. This evidence indicates that speech envelope is an auditory feature that is sufficient for speech understanding.

Given the essential role of the speech envelope in speech understanding, it is not surprising that speech envelope has been found to be represented in the human auditory system. In particular, studies that used brain recordings with high temporal resolution found that the variability in the speech envelope correlates with (i.e., is tracked by) the variability of electrical potentials and currents in human cortex [11], [12]. Furthermore, the quality of this tracking predicts the quality of speech comprehension [9].

However, it has been difficult to determine which cortical regions track this essential auditory feature. The availability of this information is a critical first step in understanding the individual stages of computations the auditory system uses to process speech-related signals. Progress in this direction is impeded by the limitations of the acquisition techniques used in current studies of the neural representation of the speech envelope. Specifically, the techniques used in current studies feature either high temporal resolution [9], [11], [12] or high spatial resolution [13]–[17], but not both.

To overcome these limitations, researchers have recently turned to electrocorticography (ECoG), an acquisition technique that combines high temporal resolution with favorable spatial resolution. Using this technique, it has been found that the speech envelope is tracked in the presumed core of human auditory cortex [18].

We asked whether and how speech envelope is tracked across human auditory cortex, outside the presumed core regions. In particular, we asked whether and how speech envelope is tracked in unisensory brain areas situated relatively early in the auditory pathway, and in higher multi- and supra-modal areas [4]. To provide an answer, we recorded neural activity using ECoG electrode grids placed on the left hemisphere of five human subjects listening to a spoken story. We found that human non-primary auditory cortex faithfully tracks speech envelope. The effect is stronger in areas situated relatively early in the auditory pathway (belt areas surrounding the auditory core) compared to higher-order regions including the superior temporal gyrus and the posterior inferior frontal gyrus.

In a supplementary analysis, we investigated whether auditory cortex also tracks sound envelope of speech-unrelated stimuli [18], [19]. To do so, we presented the subjects with a song that featured a block of singing (i.e., a different kind of speech) and a block of pure melody (no speech). We found that the envelope of singing and pure melody is tracked only in the belt areas and to a lesser degree than speech.

Together, we provide the first electrophysiological evidence that non-primary auditory cortex, in particular the cortex incorporating the belt areas surrounding the auditory core, tracks the temporal envelope of speech. To a lesser degree, this region also tracks the envelope of other naturalistic stimuli including lyrics and melody.

## Results

We recorded the neural activity of the cortex using electrocorticographic (ECoG) electrode grids placed on the left hemisphere of the cortex of five human subjects (Table 1) while they were attentively listening to a spoken story. The story was presented to each subject once, without repetition. Thus, in our study, data are not averaged across multiple stimulus presentations (typically referred to as trials in the literature).

**Table 1:** Subject profiles. All subjects had normal cognitive capacity, as assessed by the Wechsler Adult Intelligence Scale-III [45].

Subj.	Age	Sex	Handedness	Lang. dominance	Grid Locations	Channels
A	29	F	R	L	Left fronto-parietal	64
					Left temporal	23
					Left temporal pole	3
					Left occipital	6
B	30	M	R	L	Left frontal	40
					Left temporal	35
					Left temporal pole	4
					Left occipital	4
C	26	F	R	L	Left frontal	64
					Left temporal	35
					Left temporal pole	4
					Left occipital	6
D	56	M	R	L	Left frontal	56
					Left temporal	35
					Left occipital	6
E	45	M	R	L	Left fronto-temporal	54
					Left temporal pole	4

We quantified neural activity of our recordings in the high gamma range (75–115 Hz, see Methods). We focus on the high gamma range, because activity in this range has been shown to reflect multi-unit discharge rates and local field potentials of neuronal ensembles underneath each electrode [20]–[22]. Furthermore, this signal has been shown to track the envelope of speech-related sounds in the putative core auditory cortex in humans [18].

**Figure 1:**
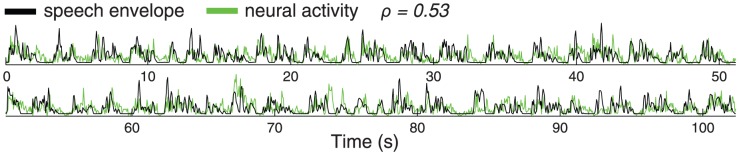
High gamma activity in human auditory cortex tracks the envelope of speech. Black: Time course of the speech envelope. Green: High gamma activity recorded by a channel positioned in the belt areas in subject C (see Figure 5) while the subject listened to a narrated story. For the visualization purpose of this figure, we graphically scaled the magnitude of the neural signal to the magnitude of the envelope signal. The Spearman correlation between the two signals is 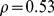.

Figure 1 shows the time course of high gamma activity of a channel located within the belt areas surrounding the auditory core (briefly, belt areas [23]), superimposed on the time course of the envelope of the spoken story (Methods). The figure demonstrates that the neural signal faithfully tracks the speech envelope (Spearman correlation 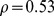). This effect is intriguing given that the channel is positioned within the belt areas–-and not implanted in Heschl's gyrus as was the case in a previous study [18]–-and given that this tracking is observed without the necessity to average neural signals over many repeated trials as is typically done in the literature.

**Figure 2:**
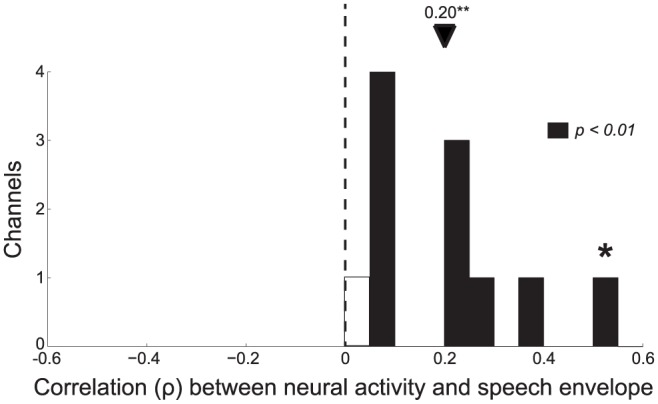
Tracking of speech envelope for each channel located in the belt areas. The Spearman correlation coefficient (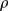) between the speech envelope and gamma activity for each channel in belt areas surrounding the auditory core. Filled bars denote the cases of significant correlation (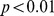). The star refers to the example channel shown in Figure 1 and Figure 5.

The effect for each channel recorded in this region (11 channels in the 5 subjects, see Methods) is given in Figure 2. The figure reveals that all (11/11) channels recorded in this region show a positive correlation. Furthermore, the correlation is significant (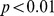) for most channels (10/11). Thus, these results demonstrate that the envelope of speech is faithfully tracked in human non-primary auditory cortex.

**Figure 3:**
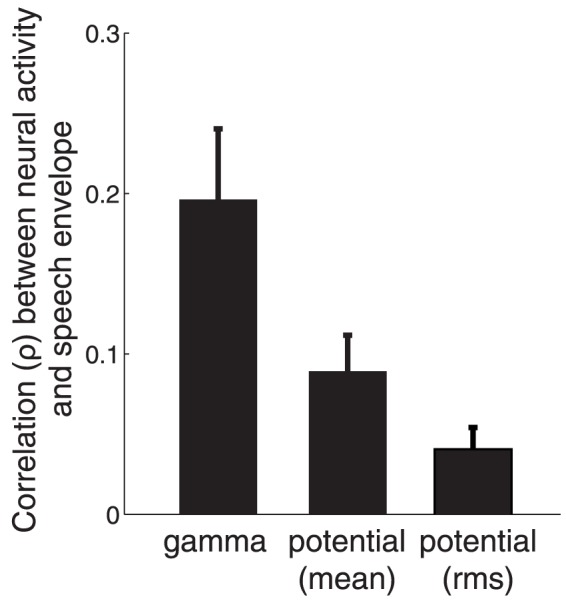
High gamma activity tracks the envelope of speech better than does the raw potential. Mean
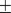
SEM Spearman correlation coefficient (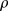) between each neural signal and speech envelope. The mean is computed across all channels in the belt areas (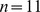).

We next tested whether the speech envelope is tracked, besides the gamma activity, also by a signal typically investigated in human studies. In particular, several studies identified correlations with signal envelope using time-locked average electric potentials or currents [9], [11], [12], [19]. We investigated the effect of the raw potential in our neural recordings (see Methods). We found (Figure 3) that high gamma activity is substantially more sensitive to the speech envelope compared to the raw potential (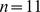, mean 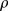
(gamma)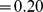, mean 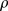
(potential)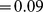, paired two-tailed t-test 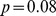 (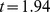, 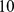 df)). The same result holds when the potential is computed as the rms value, and not as the mean–-see Methods (mean 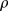
(potential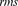)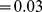, gamma versus potential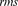 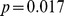 (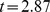, 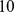 df)). Due to the superior sensitivity of high gamma activity to envelope information, henceforth, we quantify all neural effects strictly using high gamma activity, and refer to this signal shortly as “neural activity”.

Next, we investigated how the speech envelope is tracked in other cortical regions involved in speech processing. Specifically, we measured neural activity over the superior temporal gyrus (briefly, STG) and in the posterior inferior frontal gyrus (briefly, Broca's region). We focused on the STG because this region shows responses specific to intelligible speech [13], [14]. Furthermore, posterior parts of this region have been traditionally associated with speech perception [24]–[26] and, more recently, also with speech generation [5]. We focused on the Broca's region as this region has traditionally been associated with speech production [27], and it is thought to be a part of the articulatory network in recent view [4], [5].

**Figure 4:**
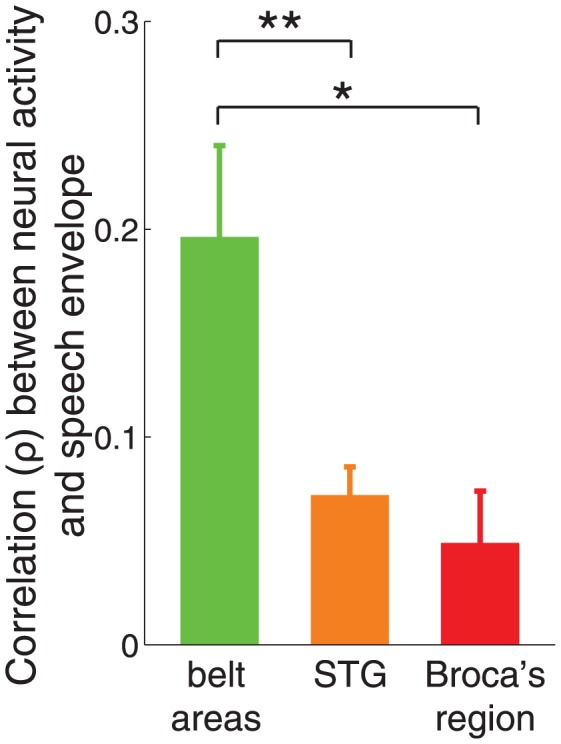
Tracking of speech envelope in three auditory cortical regions. Mean
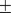
SEM Spearman correlation coefficient (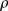) between neural activity and speech envelope in each region of interest. The mean is computed over all channels in each area (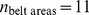, 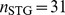, 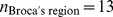). Stars denote the significance of the difference in means (two-tailed t-test), *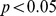, **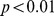.

Figure 4 compares the magnitude of the tracking of the speech envelope in these three regions of interest. The figure reveals that speech envelope is tracked predominantly in the belt areas (mean 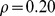, 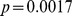 (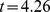), two-tailed t-test, 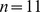) compared to the STG (mean 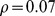, 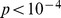 (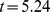), 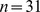) and the Broca's region (mean 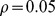, 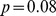 (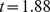), 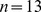, 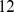 df). Importantly, the belt areas track the speech envelope significantly better than the STG (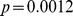 (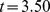), two-tailed t-test, 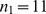, 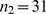) and significantly better than the Broca's region (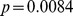, (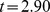), 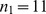, 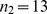). Note that the regions differ in the number of channels (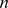). Thus, an effect of a small magnitude (a small 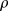) may be highly significant (a small 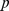) for a region with a high 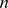 (e.g., STG).

Similar results were obtained when we further evaluated the high gamma neural activity in a broader frequency range, 70–500 Hz (belt areas versus STG: 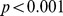; belt areas versus Broca's region: 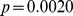; mean 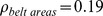, 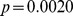; mean 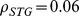, 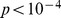; mean 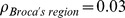, 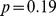). Similar results are also obtained, for the frequency range 75–115 Hz, when we use Pearson's instead of Spearman's correlation (belt areas versus STG: 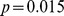, 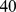 df; belt areas versus Broca's region: 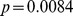; mean 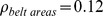, 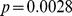; mean 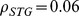; mean 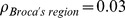).

**Figure 5:**
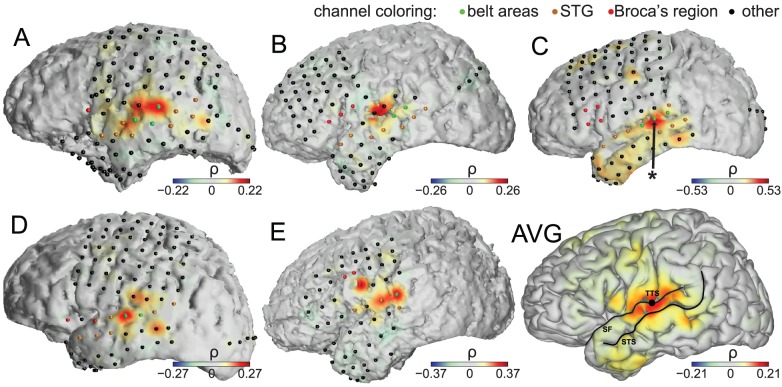
Neural tracking of speech envelope at each recording site in each subject. Color hue (see colorbars) gives 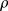 at each channel for the individual subjects (A–E), and for the subject average (AVG). Individual channels implanted in each subject are shown in green (belt areas), orange (STG), red (Broca's region), or black (other regions). The location of each channel was determined using the Talairach Atlas daemon (see Methods). In subject C, the arrow points to the channel for which we illustrated the tracking effect (Figure 1). STS: superior temporal sulcus; SF: Sylvian fissure; TTS: transverse temporal sulcus (perpendicular to the view plane).

We further validated these results by characterizing the spatial topography (see Methods) of the tracking effect (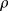) in each subject (Figure 5). This analysis confirms that speech envelope is tracked predominantly by the regions within or close to posterior parts of the superior temporal gyrus. As discussed in more detail in the Methods, our automatized procedure of co-registration of electrode locations with anatomical data may be imperfect (e.g., one channel in subject D and some channels in subject E). Nonetheless, the same principal results as those shown in Figure 4 hold when subject D (mean 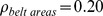, 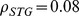, 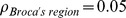; belt areas versus STG: 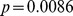, belt areas versus Broca's region: 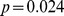, 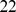 df) or subject E (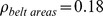, 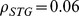, 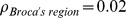; belt areas versus STG: 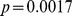, belt areas versus Broca's region: 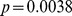) are excluded from the analyses.

**Figure 6:**
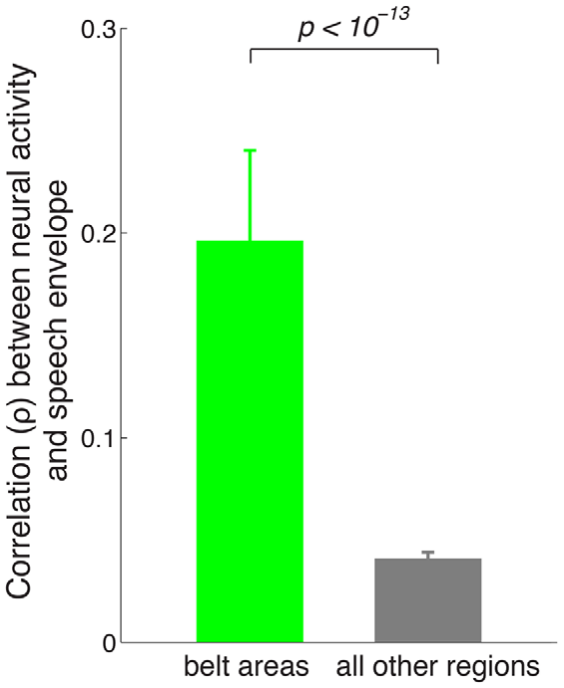
Tracking of speech envelope in early auditory regions compared to all other regions. Mean
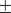
SEM Spearman correlation coefficient (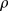) between neural activity and speech envelope in belt areas (green) and all other regions (gray). The mean is computed over all channels in each case (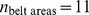, 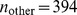).

Figure 5 shows that the tracking effect is observed more strongly in regions situated relatively early in the auditory pathway compared to other cortical regions. We quantified this impression by comparing the mean tracking effect for channels positioned within the belt areas and channels in all other regions (Figure 6). The figure reveals a highly significant difference (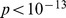, two-tailed t-test). Thus, the speech envelope tracking effect is observed predominantly early in the auditory pathway.

**Figure 7:**
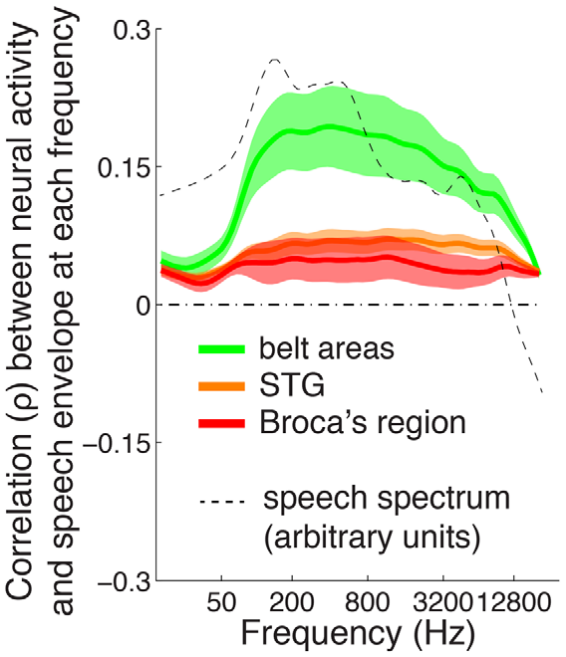
Neural tracking of speech envelope at each frequency of the sound. Mean
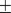
SEM Spearman correlation coefficient (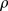) between neural activity and speech envelope for each frequency, for each region of interest. The dashed line gives the average spectrum of the speech in logarithmic units.

Both speech and animal vocalization carry information also in the frequency domain, in the form of frequency-modulated (FM) sweeps [1], [5]. Given this frequency composition of the speech signal, it is possible that each of the three speech-sensitive regions of interest is sensitive to envelope information in particular range of stimulus frequencies. We thus extracted the envelope of the speech signal at each frequency in the range from 16 Hz to 16 kHz (see Methods). We then computed the correlation (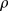) between neural activity and the envelope of speech at each frequency in this range. The result is shown in Figure 7. Two effects are observed. First, this figure confirms the result reported in Figure 4 and of Figure 5 that the speech envelope is predominantly tracked in the belt areas, and shows that this is true regardless of the frequency at which the envelope is assessed. Second, belt areas activity tracks the speech envelope starting at a sound frequency of about 100 Hz, which interestingly approximately equals the lower limit of the fundamental frequency of human utterances [1], [28].

**Figure 8:**
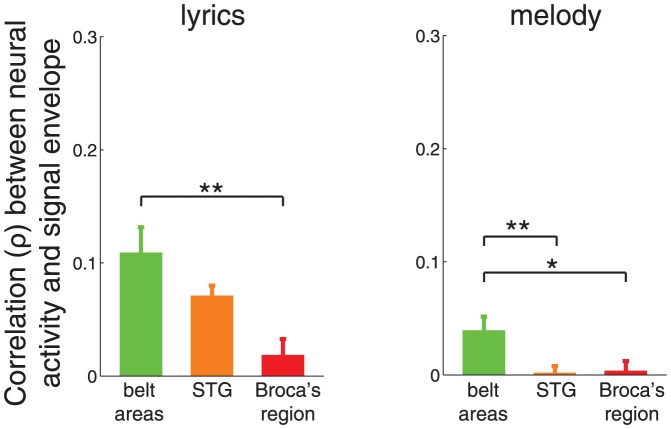
Tracking of speech-related and melodic stimuli in human cortex. Mean
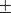
SEM Spearman correlation coefficient (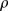) between neural activity and the speech envelope, for each region of interest. Left: stimulus containing lyrics. Right: melody. The mean is computed across all channels in each area. Stars denote the significance of the difference in means (t-test), *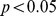, **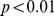.

Thus far, we have assessed the extent to which the human auditory system tracks the envelope of speech. In an additional analysis, we assessed the strength of the tracking effect also for other kinds of naturalistic stimuli, speech-related and speech-unrelated. To this end, we presented to our subjects–-besides the speech stimulus–-also a song (see Methods). We extracted from the song periods of singing (“lyrics”), and used the melodic part of the song in which no singing occurs as a speech-unrelated stimulus (“melody”). We assessed envelope tracking for these two additional stimuli the same way as we did for the speech stimulus. The result is shown in Figure 8. Two main effects are observed. First–-and in line with the observations made for the speech stimulus–-stimulus envelope is predominantly tracked in the belt areas (lyrics: belt areas versus STG, 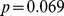 (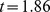), two-tailed t-test; belt areas versus Broca's region, 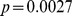 (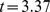); melody: belt areas versus STG, 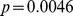 (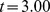), two-tailed t-test; belt areas versus Broca's region, 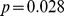 (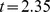, 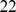 df)). Second, the envelope of lyrics and melody seems to be tracked by the considered cortical regions substantially worse than speech. We assessed these two main effects on envelope tracking (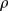) using a two-way ANOVA, with factors cortical region (belt areas, STG, Broca's region) and stimulus type (speech, lyrics, melody). Both factors had a highly significant impact on 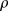 (cortical region, 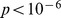 (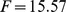); stimulus type, 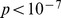 (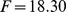)). Thus, this analysis, along with the data shown in Figure 4 and Figure 8, suggests that stimulus envelope is tracked predominantly in the belt areas, and suggests that the envelope of other types of stimuli, including lyrics and melody, is encoded relatively weakly compared to speech. Besides these findings, an important observation is that belt areas, albeit somewhat weakly, significantly track the envelope of melody (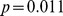, two-tailed t-test, 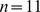, 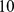 df).

The above effects could not be observed had we used–-instead of high gamma activity–-the less sensitive raw electric potential, whose time-averaged form has been employed in other studies [9], [11], [12], [19]. Using the raw potential, the two-way ANOVA with factors brain region and stimulus type fails to detect differences in envelope tracking (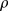) among cortical regions (main effect of cortical region, 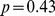 (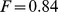)), and is weakly sensitive to stimulus type (main effect of stimulus type, 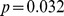 (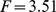, 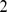 df)). This is in contrast to the sensitivity of high gamma (main effect of cortical region, 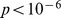 (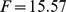); main effect of stimulus type, 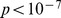 (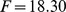)).

## Discussion

We recorded cortical responses in humans listening to naturalistic auditory stimuli, including speech and singing (speech-related stimuli), and music (speech-unrelated stimulus). We observed that high gamma activity in the belt areas surrounding the auditory core robustly tracks the envelope of speech-related stimuli. The effect is observed during a single presentation of the stimulus to each subject. We found that the tracking effect is strongest in the region incorporating the belt areas. This region tracks, besides speech-related stimuli, also the envelope of speech-unrelated stimuli (melody), albeit to a lesser degree compared to speech. Other regions involved in speech processing, including the STG and the Broca's region, track the envelope of speech-related stimuli only, and the effect in these regions is substantially weaker compared to the effect of speech in the belt areas.

These findings are consistent with the idea of hierarchical representation of speech-related sounds in the auditory system [5]. In this regard, our data show that the belt areas represent a simple acoustic feature–-envelope–-strongly, and regardless of what kind of stimulus is presented (speech-related or speech-unrelated). This suggests that the belt areas process simple acoustic features of the stimulus, and thus represent a low stage in the speech-processing hierarchy. In contrast, the considered higher-order regions (STG, Broca's region) represent envelope only weakly, and specifically for speech. This suggests that these regions are more invariant to representing simple acoustic features of the stimulus such as the speech envelope and are thus positioned higher in the speech-processing hierarchy. The idea that these regions specialize in a higher, abstract (lexical/syntactic/semantic) level of speech analysis is supported also by imaging studies [13], [14], [16], [17]. These studies have revealed that non-primary cortical regions (i.e., left middle and anterior superior temporal sulcus) show differential responses when complex sound features are compared with simple acoustic features. This holds true for both the comparison of phonetic vs. acoustic sound features [16], [17], and for the comparison of semantic vs. acoustic features [13], [14].

Notably, the finding that the considered higher-order auditory regions represent speech envelope only weakly is consistent with but does not prove the validity of the hierarchical processing model. In particular, these regions, while invariant to speech envelope or possibly not encoding speech envelope at all, may at the same time act as lower-order processing nodes for simple acoustic features of speech other than the speech envelope. This way, these putative higher-order speech processing regions may not neatly fit the hierarchical speech-processing model.

In this study, we also tested whether the envelope-tracking effect is specific to speech or whether it can be observed also for other kinds of stimuli (speech-unrelated stimuli). We found that the envelope of melody is in the considered auditory regions represented weakly compared to speech. Nonetheless, the effect does reach significance in the belt areas. This suggests that the stimulus envelope is expressed in the belt areas as an acoustic feature, not as a purely phonetic feature specific to speech. This is in a good agreement with studies that found, in regions in or close to belt areas, similar activation for both phonemic and nonphonemic sounds [16]–[18]. Furthermore, the belt areas may be sensitive to temporal information in general [29]. The weak coding of music envelope in the left cortex (all 5 subjects had a left coverage) is in line with proposals that the temporal variability in music is relatively sluggish compared to speech, and that music carries a substantial amount of information in the frequency domain [3]. It has been suggested [3] that the left auditory cortex specializes in processing of temporal information, whereas the right auditory cortex is more sensitive to information in the frequency domain. A more recent piece of evidence comes from an imaging study [30], which found that a manipulation in pitch of melodic sounds was reflected mainly in the right hemisphere, and much less in the left. Future electrophysiological studies could determine how the envelope of music is tracked in the right hemisphere.

Temporal information related to primordial forms of speech–-animal vocalization–-has been shown to be represented in discharge rates of neurons in cat and marmoset primary auditory cortex [31]–[34]. One of these studies [31] reports that about 60% of units in primary auditory cortex (A1) track the onset of a vocalization, and about 40% of A1 units track major peaks in that sound. Interestingly, the tracking of the vocalization envelope becomes particularly salient when the phase-locked multi-unit responses are summed together. Given that synchronized ensembles of multi-unit activity are correlated with high gamma power of the LFPs [20]–[22], an intriguing possibility is that the high gamma activity that we report in this study is closely related to the multi-unit discharges recorded in animal primary auditory cortex during vocalization. Indeed, it has been found that the high gamma activity can be closely tied to neuronal discharge activity in human auditory cortex when subjects listen to naturalistic stimuli [20], [22].

Most previous studies that reported tracking of speech envelope used the averaged time-locked raw electric potential [9], [11], [12], [19]. One of these studies [19] also used ECoG–-the modality we worked with in the present study. Although that study found large effects in the amplitude of cortical potentials as a function of modulation frequency, it found a relatively uniform representation of envelope information across the studied cortical regions (including primary/secondary auditory cortex, and posterior and anterior parts of superior temporal gyrus). Indeed, these results are congruent to our results when we work with raw electric potential–-this signal tracks speech envelope weakly, and its low sensitivity does not detect the differences in coding of envelope information among the individual cortical regions. Furthermore, this finding is congruent with the result of [18]. This study reports that that gamma power measured at ECoG channels implanted in Heschl's gyrus tracks sound envelope significantly more strongly than cortical potentials. However, future work shall elucidate how the results obtained using a time-locked and averaged raw electric potential in previous studies compare to the results obtained using the temporally unconstrained raw potential considered in our study.

In summary, the speech envelope is an auditory feature that is essential for speech understanding. We provide the first electrophysiological account of the tracking effect in human non-primary auditory regions. Our data reveal that the speech envelope is encoded most strongly relatively early in the auditory pathway, in particular in the belt areas surrounding the auditory core. These regions encode, to a lesser degree, also the envelope of a melody. In comparison, higher-order regions (STG and Broca's region) track the envelope of speech only, thus indicating that these regions encode speech envelope as a phonological–-not purely acoustic–-feature, and they do so only weakly. These results are in line with previous suggestions that these regions specialize in more abstract, high-level (lexical/syntactic/semantic) analysis of speech.

Looking forward, the high sensitivity of neural signals recorded using electrocorticography to temporal information in the stimulus reported in this study may serve as a powerful tool to study other fine temporal aspects of auditory processing in humans, while providing sufficient spatial detail to characterize the individual cortical regions involved.

## Methods

### Subjects

Five patients with intractable epilepsy, two women (Subjects A and C) and three men (Subjects B, D, and E), participated in this study. All subjects were left language dominant (Wada test). These patients underwent temporary implantation of subdural electrode arrays for the localization of seizure foci prior to surgical resection. Table 1 summarizes the subjects' clinical profiles. All subjects gave written informed consent through a protocol reviewed and approved by the Wadsworth Center Institutional Review Board. In all subjects, the seizure focus was localized to the anterior left temporal lobe using visual inspection of ictal ECoG signals [35]. Prior to resection, the seizure focus was delineated from eloquent auditory and language cortex using electrical cortical stimulation mapping [36]. The implanted electrode grids (Ad-Tech Medical Corp., Racine, WI) consisted of platinum-iridium electrodes, 4 mm in diameter (2.3 mm exposed) with an inter-electrode distance of 10 mm. Each subject had postoperative anterior-posterior and lateral radiographs, as well as computer tomography (CT) scans to verify grid locations.

### Auditory Stimuli

Subjects were asked to listen to a male voice narrating four fictional stories from daily life, which were part of the Boston Aphasia Battery [37]. The fictional stories were 1∶42 minutes (102 s) long, digitized at 44.1 kHz in waveform audio file format, and were binaurally presented to each subject using in-ear monitoring earphones (AKG IP 2, 12 Hz to 23.5 kHz audio bandwidth, 20 dB isolation from environmental noise). The sound volume was set to a comfortable level. The envelope of the stimulus is shown in Figure 1. The spectrum of the stimulus is shown in Figure 7. Subjects were also asked to listen to the song Another Brick in the Wall - Part 1 (Pink Floyd, Columbia Records, 1979). The song was 3∶10 minutes long. We chose this song because it features speech-related (lyrics) and speech-unrelated (melody) parts. We extracted from the song periods of singing (0∶41 minutes in total) and periods of instrumentally-carried melody (2∶29 minutes in total). The periods of singing and melody were interleaved in the first 1∶20 minutes of the song. The remaining part of the song consisted of pure melody. We obtained similar results when we considered as the melody stimulus all melody periods (2∶29 minutes in total) or only the last 1∶50 minutes of the song (continuous segment of pure melody). Thus, we used all melody periods. Each stimulus was presented to each subject once and only once. Thus, in our study, data are not averaged over multiple stimulus presentations (referred to as trials in the literature).

### Extraction of Sound Features

We extracted the envelope of a given stimulus by computing the power of the raw sound signal in each time window (consecutive windows of 50 ms duration, no overlap). The length of the analysis window (50 ms) was chosen as short enough to capture the variation in speech envelope, and long enough to allow for meaningful estimation of the gamma component of the neural signal. An example result for the speech stimulus is given in Figure 1. Separately, for the purpose of Figure 7, we further extracted the envelope at each frequency of a given stimulus. To do so, we computed spectral power, for each frequency, of the raw sound signal in each time window. To compute spectral power, we used the fast Fourier transform. (Some studies used the Hilbert transform [9], [18] for the same purpose. We opted for the Fourier transform, as in general this method has been the prevalent method when extracting frequency information from sound signals.)

### Electrophysiological Recording

Data collection and stimulus presentation were realized using the general-purpose software BCI2000 [38], [39] and g.USBamp biosignal acquisition devices (g.tec Medical Engineering, Schiedlberg, Austria). The g.USBamp devices amplified the ECoG signals, low-pass filtered them at 5000 Hz, digitized them at 38400 Hz, and finally downsampled the result to 1200 Hz. The downsampling step preceded all analyses performed in the paper. Electrodes that clearly did not contain ECoG activity (e.g., due to broken wires, reference location, etc.) were excluded from our analyses (subject A: 1 channel, B: 1, C: 1, D: 2, E: 2).

### Cortical Mapping

We used the software package Curry (Neuroscan Inc., El Paso, TX) to create subject-specific 3D cortical brain models from high resolution pre-op MRI scans, and to co-register the MRIs with post-op CTs and extract the stereotactic coordinates of each grid electrode. We acquired two sets of T1 weighted MRI scans, a sagittal one to define the origin of the coordinate system (i.e., anterior/posterior commissure), and a coronal one to reconstruct the cortical surface. Both scans were acquired on a 1.5 Tesla General Electric MRI scanner with 3 mm and 1 mm slice thickness for the sagittal and coronal scans, respectively. The anatomical and functional (Brodmann) areas of each channel were assigned using the Talairach Atlas daemon [40] (http://www.talairach.org/daemon.html). Using this procedure, we identified 11 channels in the belt areas surrounding the auditory core (“belt areas” [23], BA 42, extending to planum temporale, and possibly including the parabelt), subject A: 3 channels, B: 2, C: 3, D: 2, E: 1), 31 channels in BA 22 extending over the superior temporal gyrus (“STG”), and 13 channels in BA 44/45, a part of the posterior inferior frontal gyrus (“Broca's region”). All channels in a given region were included in the analyses, i.e., we do not restrict our analyses solely to channels that show a significant relationship to stimulus envelope. We projected the electrode coordinates onto the reconstructed brain models and generated activation maps using a custom program (jan@eye-hand.wustl.edu) written in Matlab (The Mathworks Inc., Natick MA). Activations were smoothed using a linear kernel falling from 1 to 0 over the distance of 10 mm. In Figure 5 AVG, we plotted neural activations that were averaged over all subjects on a template cortical (pial surface) model. We obtained this model from the source code provided on the AFNI SUMA website (http://afni.nimh.nih.gov/afni/suma). The electrode coordinates for each subject were projected on this model and average activations were computed using the same custom Matlab program. Although the brain model of each subject was expressed in the same Talairach space as the generic AFNI model, each individualized brain model slightly differs from this generic model. The average activation shown in Figure 5 AVG should thus serve only an illustrative purpose. Notably, the electrode locations are determined by lateral skull radiographs to identify the stereotactic coordinates of each grid electrode using automated software [41]. This procedure inherently leads to several millimeters of variance in the coordinate estimates [41]. At the same time, the extent of each cortical regions considered in this study was much greater than this expected localization variance. Furthermore, using this procedure, we detected significant differences in the envelope tracking neural effect across the considered regions. Thus, the relatively small variance in the estimation of stereotactic coordinates does not affect our principal results.

### Extraction of Neural Activity

We first pre-processed the raw ECoG signals using a common average reference (CAR) spatial filter (as in [42], [43]). To extract the high gamma activity, we converted the time-series ECoG data into the frequency domain by applying an autoregressive model of order 12 (a different value has minimal effects on the results) to each of the 50 ms time windows. We averaged the obtained spectral amplitudes in the high gamma (75–115 Hz) frequency range. We used this frequency range to match the range of a previous study [43]; this range avoids the frequency of the line noise (60 Hz) and its harmonics (120 Hz, 180 Hz, etc.). Similar results (see Results) are obtained when we used a broader frequency range, 70–500 Hz. Besides activity in the high gamma band, we also computed the raw unrectified ECoG potential, which has previously been shown to correlate with different aspects of motor function [42]–[44] and labeled local motor potential (LMP), by averaging the raw time-series ECoG samples in each of the 50 ms windows. Notice that the raw potential is a purely time-domain signal, whereas the high gamma signal represents the evolution of the high gamma amplitude over time. Both signals are extracted (in 50 ms windows) from the same raw time-series ECoG samples. For completeness, we computed the potential also as a root-mean-square (rms) value, instead of the mean, over each window. The same principle results hold, including the same frequency profiles (Figure 7), when we evaluated our data in 100 ms instead of 50 ms windows.

### Assessment of Envelope Tracking

We quantified the relationship between neural activity and stimulus envelope by computing the Spearman correlation 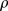 between these two quantities. To account for the temporal lag between these two quantities, we computed this correlation for each lag between the two signals in the range 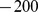 ms to 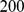 ms, in 10 ms steps. Throughout the paper, we report the maximum value of the correlation over this range. The time of this maximum represents the optimal lag between neural activity and stimulus envelope. We estimated the average value of this lag for all channel-envelope pairs that were at least weakly correlated (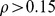) to ensure that an optimal lag could in principle be found. Neural activity lags, on average, over all stimuli and all frequencies, behind stimulus envelope by 88.6
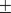
3.5 (mean
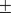
SEM) ms in the belt areas, 89.9
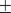
5.9 ms in the STG, and 86.7
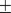
3.0 ms in the Broca's region. Notice that these values are potentially not precise, and thus no conclusions should be drawn based on these values. A more precise value of the lag should be determined in a separate study, by removing the autocorrelation structure in the stimuli, and by carefully comparing only those channels in each area that encode envelope similarly strongly. Throughout the study, 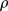 values are Fisher-transformed prior to any test.

The nonparametric Spearman's statistic gave similar results (see Results) as the parametric Pearson's. We used the Spearman's statistic because it is potentially more robust and has fewer assumptions about signal properties than the Pearson's statistic.

To assess the relationship between neural signals and the sound signal, we calculated, for each location in a particular region, the correlation coefficient between the sound signal and the neural signal. We then asked whether this distribution of correlation coefficients is significantly different from zero (see Results). Using this measure, we are able to obtain both a highly significant effect (e.g., for speech in the belt areas, mean 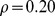, 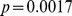, 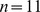), as well as no effect that serves as the negative control (e.g., for melody in the STG, mean 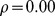, 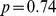, 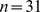, or for speech in the Broca's region, mean 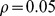, 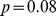, 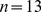).
